# Fatherhood and Sperm DNA Damage in Testicular Cancer Patients

**DOI:** 10.3389/fendo.2018.00506

**Published:** 2018-09-13

**Authors:** Donatella Paoli, Francesco Pallotti, Andrea Lenzi, Francesco Lombardo

**Affiliations:** Laboratory of Seminology-Sperm Bank “Loredana Gandini”, Department of Experimental Medicine, “Sapienza” University of Rome, Rome, Italy

**Keywords:** sperm DNA damage, sperm chromatin, testicular cancer, reproductive outcome, fatherhood, cancer survivors

## Abstract

Testicular cancer (TC) is one of the most treatable of all malignancies and the management of the quality of life of these patients is increasingly important, especially with regard to their sexuality and fertility. Survivors must overcome anxiety and fears about reduced fertility and possible pregnancy-related risks as well as health effects in offspring. There is thus a growing awareness of the need for reproductive counseling of cancer survivors. Studies found a high level of sperm DNA damage in TC patients in comparison with healthy, fertile controls, but no significant difference between these patients and infertile patients. Sperm DNA alterations due to cancer treatment persist from 2 to 5 years after the end of the treatment and may be influenced by both the type of therapy and the stage of the disease. Population studies reported a slightly reduced overall fertility of TC survivors and a more frequent use of ART than the general population, with a success rate of around 50%. Paternity after a diagnosis of cancer is an important issue and reproductive potential is becoming a major quality of life factor. Sperm chromatin instability associated with genome instability is the most important reproductive side effect related to the malignancy or its treatment. Studies investigating the magnitude of this damage could have a considerable translational importance in the management of cancer patients, as they could identify the time needed for the germ cell line to repair nuclear damage and thus produce gametes with a reduced risk for the offspring.

## Introduction

Spermatogenesis is the process through which undifferentiated stem cells proliferate and differentiate into spermatozoa. It takes place in the seminiferous tubules in the testicles and is classically divided into three stages: spermatogonial proliferation, meiosis, and spermiogenesis (1, 2).

The mature spermatozoon's main function is to transfer the undamaged haploid genome to the oocyte. During the spermatogenetic process the protection of DNA is thus of considerable importance, and it is kept safe through its sperm-specific packaging (3). This is made possible by the binding of DNA with protamines (4–11), which collapse into a toroidal structure and anchor to matrix-associated regions (12–14). Correct DNA-matrix structure is required to replicate male pronuclear DNA and control nuclear integrity after fertilization, since the nuclear matrix plays a pivotal role (15–17). This specific chromatin organization is associated with the recruitment, integrity and function of DNA repair components (18).

DNA strand breaks may occur as a natural consequence of chromatin reorganization, and sperm with highly condensed chromatin may also suffer damage. This process begins in the nuclear matrix. The action of external nucleases causes DNA to be degraded and released by this structural scaffold (19, 20). The DNA fragmentation process begins during epididymal maturation and transit through the vas deferens. The luminal fluid contains enzymes which act like nucleases, activating an apoptosis-like mechanism that in turn triggers sperm self-destruction if the sperm cell is damaged for some reason. Various types of DNA strand breaks have been identified: (a) single-strand breaks (SSBs) are probably also generated by reactive oxygen species released from the mitochondria in damaged sperm (21–23); (b) reversible double-strand breaks (DSBs) are probably generated by the action of topoisomerase II (19, 24); and (c) non-reversible DSBs are induced by the action of nucleases that enter from the luminal fluid (18, 19, 25, 26).

These alterations may be induced by the dysregulation of normal apoptotic mechanisms and/or by a rise in oxidative stress due to pathological or iatrogenic factors. The apoptotic process is a control system for the overproduction of male gametes (27–29) through which endonucleases induce the formation of double-strand nicks in the DNA with subsequent DNA degradation, chromatin condensation and the formation of apoptotic bodies (30–33).

The sperm cell has three options: (a) repair the damage, (b) activate the apoptotic process, causing cell death, or (c) tolerate the damage, resulting in mutations which could be transmitted to future generations (2, 34, 35). However, mature sperm are incapable of repairing DNA damage, as translation and transcription activities are silenced in the later stages of spermatogenesis (34).

Some authors have shown in animal models that the spermatocyte can actuate various DSB repair mechanisms (36) through non-homologous end-joining (NHEJ) and homologous recombination (HR) (35, 36). These two mechanisms may co-exist (36–38) but if inadequate, can introduce new mutations. Despite all this, sperm with fragmented DNA can be fertile, and the biological impact of an abnormal sperm chromatin structure depends on the combined effects of the extent of DNA or chromatin damage in the sperm and the capacity of the oocyte to repair that damage (35, 39–42).

### Clinical implications

From a clinical perspective, sperm DNA damage, including chromatin fragmentation, has been associated with impaired spermatogenesis and infertility and can have negative consequences on the reproductive process (43–46), including recurrent pregnancy loss (RPL) (47). Various studies have investigated the relationship between sperm DNA damage and reproductive outcome. Studies of natural fertility highlighted that sperm DNA damage is associated with a prolonged time to pregnancy (48) as well as a low probability of achieving natural pregnancy (49). Several studies also reported an association between low pregnancy rates in ART and DNA damage (50). Moreover, various authors found a significant correlation between DNA fragmentation and pregnancy loss after IVF or ICSI (OR 2.37) (51–53). Available data do not permit any correlation to be established between chromatin integrity and reproductive outcome, although there is a significant correlation between fragmentation and pregnancy loss following IVF or ICSI (54, 55), as revealed in a meta-analysis (52). Stratification of the studies by method used to analyse DNA fragmentation produced different results, with a stronger association found for TUNEL (53, 56). Most studies using TUNEL reported a significant impact on embryo development, blastocysts and pregnancy loss for both IVF and ICSI, whereas studies using SCSA obtained more variable results (57). This may be because the different methods identify different aspects of DNA damage: in fact, the alkaline Comet and TUNEL assays can directly measure the level of sperm DNA damage, while SCSA indirectly measures the susceptibility of DNA to damage, consequently influencing the discovered associations with ART outcome (58, 59).

**Figure 1:**
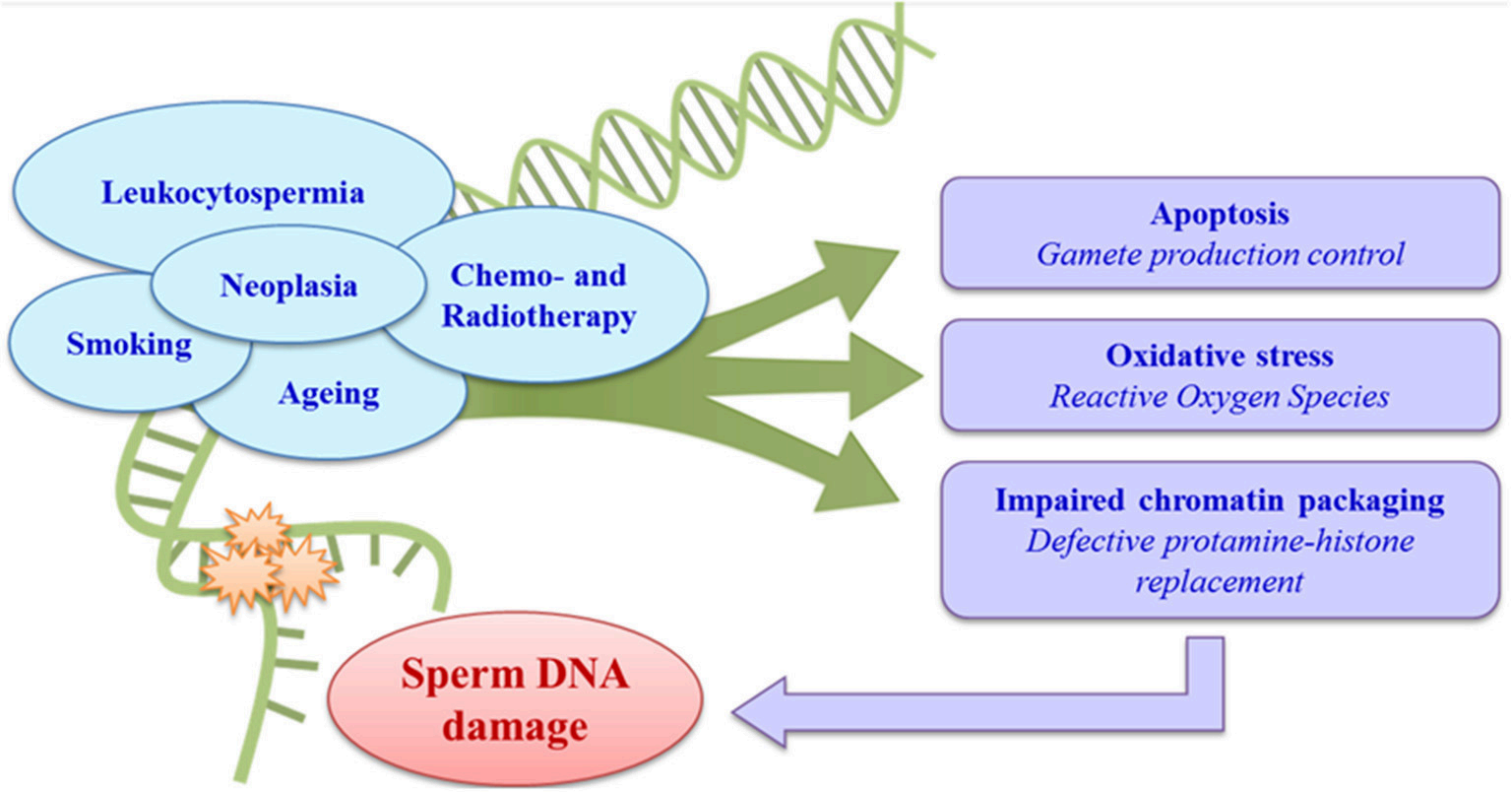
Aetiology and pathogenesis of sperm DNA damage.

Different factors, especially leukocytospermia (60, 61), smoking, obesity and other lifestyles (62–64), age (65, 66), male accessory gland infections (67), varicocele (68), and neoplastic diseases (69–72), may be correlated with increased sperm DNA damage, with a consequent impact on male fertility. Iatrogenic causes, above all the chemo- and radiotherapies used to treat cancer, can also have effects on spermatogenesis and consequently on the sperm chromatin (43, 73, 74) (Figure 1).

Cancer itself also has an important role in male infertility, with both direct and indirect effects on spermatogenesis. Particular attention has been paid to the effects of testicular cancer, the most common cancer in men of reproductive age (75), on sperm DNA. The incidence of TC varies considerably in different countries and in different ethnic groups, possibly in relation to both environmental factors and genetic susceptibility, as hypothesized in so-called testicular dysgenesis syndrome (TDS) (76–80). Various studies have investigated the association between the presence of persistent environmental pollutants in serum and the risk of TC, but no strong association has been identified (81–88). Studies of testicular cancer (70, 71) revealed impaired sperm chromatin integrity even before any antineoplastic treatment, with an increase in damaged DNA and a negative correlation between chromatin damage and semen quality (69). Despite accounting only for about 1% of all male cancers, TC was once the main cause of death from cancer in men of reproductive age, but advances in chemotherapy (CT) and radiotherapy (RT) combined with surgical techniques have produced a marked improvement in the prognosis and survival of these patients (75, 89–92). However, treatment protocols may have long-term effects including metabolic syndrome, vascular and cardiac damage, secondary cancers, and infertility (93, 94). There is also great concern about their effects on semen quality and sperm chromatin integrity, as the high cell renewal rate of the seminiferous epithelium makes it highly sensitive to these treatments (72, 95, 96). The target of any antineoplastic treatments is DNA, which becomes fragmented, leading to cell death (72, 95).

To date, there is little literature information on the damage suffered by sperm DNA after exposure to antineoplastic treatments. Above all, the duration, extent and biological significance of their effects on chromatin integrity and the time necessary to repair such damage are not yet known. The aim of our paper is to review the impact of TC and its treatment on the sperm chromatin quality and reproductive outcome of cancer survivors following both natural pregnancy and assisted reproductive technology (ART). This information has translational relevance for the management and effective counseling of these patients with regard to their reproductive potential.

## Methods

We conducted a review of the literature to evaluate the relationship between testicular cancer, sperm DNA damage and fertility. We searched the Medline (Pubmed) database using the following search terms: “sperm DNA damage AND testicular cancer,” “testicular cancer AND sperm DNA integrity after therapy,” “assisted reproduction AND testicular cancer,” “pregnancy outcome AND testicular cancer,” “fatherhood AND testicular cancer.” Additional studies were identified from the study reference lists. Only full-length articles published in English between 1986 and 2018 were searched. With regard to sperm DNA integrity, we considered only the most common methods (TUNEL, Comet, SCSA). We found 17 studies that evaluated pre-therapy sperm DNA integrity in TC patients and 11 that investigated sperm DNA damage induced by antineoplastic therapies in such patients. We also found 10 studies derived from population-based surveys or national cancer registers and 37 suitable studies with information on the outcomes of both natural and ART-derived pregnancies in TC patients.

## Chromatin integrity evaluation

Numerous methods have been developed to evaluate sperm DNA integrity, with the aim of establishing the degree of chromatin condensation. These tests have been developed in parallel with advances in ART and the increased understanding of the importance of chromatin integrity in this context (97). The most widely used methods for investigating sperm chromatin integrity in testicular cancer are SCSA, TUNEL and Comet assay.

- SCSA: The sperm chromatin structure assay (SCSA) is a cytofluorometric technique which indirectly assesses DNA strand breaks and protamine-histone replacement defects by measuring the resistance of sperm chromatin to the action of denaturing agents using the dye acridine orange (98, 99).
- TUNEL: Terminal deoxynucleotidyl transferase UTP-driven nick end labeling, or TUNEL, detects the endogenous DNA strand breaks in sperm through the enzyme TdT (terminal deoxynucleotidyl transferase). It enables the incorporation of deoxyuridine triphosphate (dUTP) in DNA fragments deriving from single or double strand breaks to be quantified by fluorescence microscopy or cytofluorimetry (100–102).
- COMET ASSAY: Single cell gel electrophoresis (SCGE), or Comet assay, enables DNA integrity to be evaluated by visualizing strand breaks in individual cells (103). The DNA from the nucleus of any damaged cells forms a comet pattern with a fluorescent head and a tail whose length and fluorescence is proportional to the number of DNA strand breaks. The analysis is performed using a fluorescence microscope with imaging software. This test can be performed in both alkaline and neutral conditions: alkaline Comet reveals both SSBs and DSBs in sperm DNA, while neutral Comet is capable of selectively detect DSBs (104). The Comet assay has been used for both the *in vitro* evaluation of the mutagenic activity of various chemicals on sperm (105) and in preliminary studies of the correlation between fertility and basal sperm DNA damage (106).

## Sperm DNA damage in testicular cancer

### Pre-treatment

Evenson et al. were the first to demonstrate abnormal sperm chromatin condensation, for up to 1 one year post-orchiectomy and before any antineoplastic treatment, in a study of 14 TC patients (107). Fossa et al. then found a significant increase in the percentage of non-condensed haploid sperm cells in 85 testicular cancer patients post-orchiectomy and pre-treatment compared with a control group. In the following years, the refinement of methods to study chromatin integrity led to increasing interest in sperm DNA damage, especially in testicular cancer, which involves the sperm production site (108). However, the actual results are hard to interpret as the available methodologies reveal different types of nuclear damage.

Although several methods claim to identify “fragmented DNA,” it is important to understand what each one is actually measuring. Some tests, such as the alkaline Comet assay and TUNEL, identify double- and single-strand DNA breaks, while SCSA assesses the susceptibility of DNA to denaturation, indirectly revealing possible strand breaks or protamine-histone replacement defects (98–106).

The theory is that DNA denaturation takes place much more easily in sites affected by single- or double-strand breaks (109, 110). This information is important in assessing the possibility of repair, as the oocyte is capable of repairing small numbers of SSBs, but DSBs may be more problematic (111, 112). Recently, Comet assay has been performed under neutral condition. This method could provide more information about the DNA breaks, although the available evidence is still limited.

It should also be remembered that strand breaks occur naturally during DNA supercoiling and relaxation (17–19, 23, 26). For this reason, we are presenting the published studies on the basis of the different methods used to assess sperm chromatin integrity in testicular cancer.

#### SCSA

Of the 11 literature reports using this method, seven found sperm DNA damage in TC patients post-orchiectomy and pre-therapy, while four did not find any difference between TC patients and healthy, fertile controls (Table 1). One of the first studies, dating back to 1997, found abnormal sperm chromatin in a small caseload of TC patients post-orchiectomy and pre-treatment compared to healthy semen donors (113). Similar results were reported by later studies which compared TC to healthy and/or fertile controls (69, 71, 119, 117, 116, 121) (Table 1). TC patients showed similar sperm nuclear damage to infertile subjects. Said et al. in particular found sperm DNA fragmentation levels to be 2-fold higher in TC patients than in healthy fertile controls (117). Stahl et al. also found greater sperm DNA damage (17.5%) in TC patients in comparison with a fertile age-matched population (116). However, the percentage of sperm with fragmented DNA fell into a moderate level of sperm DNA damage considered compatible with achieving pregnancy (125). O'Flaherty et al. also found high mean DFI and low chromatin compaction prior to chemo/radiotherapy in TC patients in comparison with healthy subjects (119). Finally, Bujan et al. found higher levels of DFI in TC patients than in controls (121).

**Table 1:** Studies of sperm DNA damage in testicular cancer patients after orchiectomy and pre antineoplastic treatment, subdivided by methodology.

**References**	**N. TC pts**	**Controls**	**Pre-treatment DNA damage TC vs. Controls**
**SCSA**			
Fossa et al. (113)	39	18 semen donors	Increased
Kobayashi et al. (69)	20	12 healthy fertile	Increased
Stahl et al. (114)	20	278 military conscripts	NOT increased
Stahl et al. (115)	25	278 military conscripts	NOT increased
O'Flaherty et al. (71)	15	21 infertile + 21 healthy volunteers	Increased
Stahl et al. (116)	25	137 healthy fertile	Increased
Said et al. (117)	39	20 healthy fertile	Increased
Smit et al. (118)	52	22 healthy fertile	NOT increased
O'Flaherty et al. (119)	16	11 infertile + 11 healthy volunteers	Increased vs. healthy
McDowell et al. (120)	37	35 healthy volunteers	NOT increased
Bujan et al. (121)	53	257 fertile	Increased
Total	341		
**COMET**			
O'Donovan et al. (122)	13	14 healthy fertile	Increased
O'Flaherty et al. (71)	15	21 infertile + 21 healthy volunteers	Increased
Kumar et al. (104)	19	20 semen donors	Increased
Total	47		
**TUNEL**			
Gandini et al. (101)	30	23 healthy + 29 infertile	Increased vs. healthy
Spermon et al. (123)	22	13 healthy	Increased
Stahl et al. (115)	19	24 military	NOT increased
Ribeiro et al. (124)	48	50 healthy fertile	NOT increased
O'Flaherty et al. (71)	15	21 infertile + 21 healthy volunteers	Increased vs. healthy
Bujan et al. (121)	53	257 fertile	NOT increased
Total	187		

In contrast, Stahl et al. investigated post-orchiectomy TC patients prior to further treatment in two studies in 2004 and 2006, finding no differences in the percentage of sperm with DNA damage between cancer patients and the control group (114, 115). Smit et al. also found no increase in pre-therapy DNA damage compared with fertile men. Moreover, sperm DNA fragmentation does not seem to be correlated with TC histotype: no significant increase in sperm DNA fragmentation was found between seminoma and non-seminoma patients (118). McDowell et al. also failed to find any significant difference in TC patients, who had a mean of 8.73% sperm DNA damage compared with the 9.88% seen in men presenting for altruistic sperm donation (120).

In conclusion, the majority of studies found increased, but moderate, levels of sperm DNA damage in TC patients compared to healthy controls. Infertile patients used as the control population may have comparable levels of chromatin damage to TC patients, supporting the hypothesis that TC could be a cause of temporary infertility by affecting semen quality.

#### TUNEL

Six studies used TUNEL. Of these, three found more sperm DNA fragmentation in TC patients while the other three did not find any differences in chromatin integrity between TC patients and healthy controls (Table 1). Gandini et al. found a significant increase in apoptotic sperm DNA fragmentation in the semen samples of TC patients post-orchiectomy and in infertile patients with oligoasthenoteratozoospermia compared with a control group of healthy men (101). This might suggest that major sperm DNA damage is one of the characteristics of spermatogenetic failure and that high apoptotic fragmentation is correlated with impaired semen parameters. In fact, this study found a negative correlation with sperm motility and a positive correlation with abnormal forms, predominantly affecting the head, in TC patients. Increased sperm DNA fragmentation in TC patients was also confirmed in a study by Spermon et al. (123). In these patients, neoplastic proliferation seems to induce intratesticular damage of the apoptosis control system. This seems to be augmented by the clinical stage of the disease. O'Flaherty et al. evaluated poor sperm chromatin quality, in terms of an increase in single- and double-strand breaks and decrease in the protamine level and chromatin compaction, in advanced TC patients against control subjects (71). This study found a non-significant trend increase in TUNEL-positive sperm in TC patients. Chromatin damage was also found in 37% of normozoospermic TC patients; interestingly, this damage was comparable to that seen in the infertile group, as also found in other studies, demonstrating that TC could be a cause of transient qualitative sperm damage, even in the absence of significant quantitative changes.

However, Stahl et al. (115), Ribeiro et al. (124), and Bujan et al. (121) did not find any correlation between TC and sperm DNA fragmentation. Stahl et al. (115) investigated TC patients, finding a similar percentage of sperm DNA fragmentation to that observed by Gandini et al. (101); the two studies differed in the control group, which in Gandini's study consisted of healthy normozoospermic subjects and in Stahl's comprised subjects with various seminal characteristics, including oligozoospermia. Ribeiro et al. also found no differences in apoptotic DNA fragmentation between patients with non-seminoma or seminoma and fertile men (124). The authors identified a mean of 12.6% sperm cells with apoptotic DNA fragmentation in the control group, 12.2% in the non-seminoma group and 12.5% in the seminoma group. In contrast with the previous studies, the authors suggested that as all patients were studied after orchiectomy, removal of the affected testis may have annulled possible effects to the remaining normal testis. Finally, Bujan et al. evaluated sperm DNA damage in the semen samples of 53 TC patients, finding no difference in the percentage of sperm DNA fragmentation between patients and controls (121).

In conclusion, half of the studies investigating the sperm of TC patients found an increase in sperm DNA fragmentation, with the authors suggesting disruption of the apoptotic control equilibrium as a possible response to the disease. It is possible that the two studies finding no differences in sperm chromatin integrity between cases and controls was because they used control groups with heterogeneous semen parameters, while the third found no differences with TUNEL, but only with SCSA.

#### Comet assay

Just three studies to date have used the alkaline Comet assay. All were performed on small caseloads and found more sperm damage, expressed as the percentage of cells with DNA forming a comet pattern, in patients than in controls (Table 1). O'Donovan investigated chromatin integrity pre- and post-therapy in various neoplastic diseases, including testicular cancer (122). This was a pilot study on a small caseload of cancer patients, including TC and fertile men. The author found a higher level of sperm DNA fragmentation in TC patients than in the controls. This finding was confirmed by O'Flaherty et al. in patients with advanced testicular cancer, for whom semen phenotype, hormone profile and genome integrity were investigated (126). The authors found impaired semen parameters (sperm concentration, normal forms, and motility), an elevated FSH serum concentration and elevated sperm DNA damage in the TC patients in comparison with the control group of healthy volunteers. More recently, Kumar et al. detected increased sperm DNA damage in 19 testicular cancers patients vs. 20 semen donors with both alkaline and neutral COMET, with higher levels of sperm DNA fragmentation in TC patients with abnormal semen parameters (104). In particular, under neutral conditions, the Authors reported a significant difference in DNA DSBs in men presenting with testicular cancer compared to fertile donors.

In summary, the literature comprises 17 papers that used various methods to evaluate sperm DNA integrity post-orchiectomy and pre-therapy in testicular cancer patients. Three used TUNEL only, eight SCSA only and three Comet assay only, while three used both SCSA and TUNEL to investigate the same caseload. Eleven studies found a difference in sperm DNA damage between TC patients and controls using one or two methods, five found no such difference while just one found different results with different methods (Table 1).

These studies have various limitations, above all the limited caseloads and use of different control groups, which could affect the comparison of the cases and controls. The use of normozoospermic or fertile subjects as the control group should undoubtedly produce a low percentage of sperm DNA fragmentation, while a control group of unselected subjects from the general population might have a slightly higher DFI. Furthermore, not all studies reported age (an important factor associated with chromatin fragmentation), histotype or clinical stage, and many did not report the clinical features. It was therefore not possible to perform a multivariate analysis to identify the various parameters that might affect chromatin integrity.

Most of the studies cited in this section found elevated sperm DNA damage in TC patients. This could be due to a maturation defect during spermatogenesis in the remaining testicle after orchiectomy, but also to the impact of stress factors, abnormal hormone production (oestrogens, human chorionic gonadotropin) or other factors linked to the development of testicular dysplasia (70, 101, 127). Testicular cancer can in fact be hormonally active, with the production of β-human chorionic gonadotropin (βhCG) and α fetoprotein (AFP), and has both local and systemic effects, including temperature changes and metabolic effects (128). Carcinogenesis may result in a systemic inflammatory state and the secretion of metabolically active cytokines can lead to damage to the germinal epithelium. Cytokines such as interleukin 1, interleukin 6, tumor necrosis factor α, and interferon γ may affect the hypothalamic-pituitary-gonadal axis (128, 129). Finally, cancer can itself be associated with malnourishment, leading to deficiencies in the vitamins and trace elements needed for optimal gonadal function, as well as psychological issues such as anxiety and depression. For these reasons, cancer itself may contribute to both quantitative and qualitative impairment of spermatogenesis, even if the literature data are contradictory.

### Post-treatment

Antineoplastic therapies are an important cause of sperm DNA damage. Interest in the toxic effects of these therapies on embryonic development has generally focused on the mother, while the paternal aspect has often been underestimated. Few studies have investigated male-mediated teratogenicity (130–132), but above all the little information that is available provides conflicting evidence of the sperm chromatin damage induced by these treatments.

Radiotherapy and chemotherapy have a damaging effect on reproductive function through both cytological and molecular effects. The negative effects include impaired spermatogenesis, resulting in oligozoospermia or azoospermia (72, 133), and an increase in aneuploidies for up to 18–24 months after the end of the therapy (134–136). Most chemotherapeutic agents are cytotoxic for cells in a given phase of the cell cycle. Furthermore, the testicle is one of the most radiosensitive tissues and is vulnerable to damage from both direct radiation or, more commonly, the scattering of radiation during the treatment of adjacent tissues (137).

While the main aim of treatment must of course be to cure the cancer itself, the future quality of life of TC patients must not be overlooked, given the now excellent survival rates. In young adults, it is important to evaluate any reproductive problems that might arise following treatment; in particular the study of sperm chromatin integrity, which could be an infertility factor or even be associated with genome instability, with consequent repercussions for any offspring.

The introduction of ICSI has significantly improved the opportunity for paternity in TC patients. However, there is a concern that it might increase the risk of transmitting defective paternal genomes to the offspring. For this reason, data concerning pre- and post-treatment sperm chromatin integrity in cancer patients, and especially TC patients, would have significant translational relevance in the management of these patients, as they could be provided with adequate counseling on their future reproductive chances. Literature reports of the impact of antineoplastic treatments give conflicting results, due to the small caseloads, the different treatments investigated and above all the different methods used to study sperm DNA damage. For this reason, as in the section above, we will present the data according to the method used.

#### SCSA

The impact of cancer treatment on sperm DNA integrity has been investigated by SCSA in 7 longitudinal studies (Table 2). There is general accordance among these studies about the negative impact of radiotherapy (43, 114, 115, 118, 121). Stahl et al. reported a significant but transient increase in DFI in the first 2 years after radiotherapy, which normalized 3–5 years after the end of the treatment (114), confirming these data in a later study with a larger caseload (115). According to Smit et al. RT has a higher impact on DFI than CT alone; after a follow-up of 0.5–3.3 years (median 1.1) this study found a significant increase in DFI in patients who had undergone RT or RT+CH compared to those who underwent chemotherapy alone (118). A multicentre study with 24 months' post-treatment follow-up found reduced chromatin compaction 6 months after the end of radiotherapy (121). Paoli et al. confirmed these observations in a larger caseload, reporting that RT-induced DNA damage increased up to 6 months post-RT, with a subsequent reduction at 12 and 24 months (43). Evidence of the effects of chemotherapy is more contradictory. Several studies did not find any differences in DNA damage post-chemotherapy (116, 118, 121). Others even found an improvement in sperm chromatin integrity: in 2004 and 2006, Stahl et al. reported that patients undergoing 1–2 chemotherapy cycles had a significant reduction in DFI at 6 and 12–24 months from the baseline, while advanced stage patients treated with more than two chemotherapy cycles showed a reduction in DFI 5 years after the end of the treatment (114, 115). The authors interpret this surprising result as a consequence of germ cell vulnerability to chemotherapy, thus causing the prevalent elimination of spermatogenic cells with DNA damage. In other words, chemotherapy could induce the removal of a subpopulation of abnormal germ cells. In contrast, in a small caseload of patients with advanced testicular cancer after orchiectomy, O'Flaherty et al. reported higher DNA damage and lower chromatin compaction prior to therapy, persisting for the entire follow-up period, in comparison with controls (119). Paoli et al. whose results in relation to radiotherapy are reported above, also found increased CT-induced DNA damage at 6 months with a more marked reduction than seen with RT at 12 and 24 months post-therapy, indicating a clear improvement in the chromatin profile at these time points (43). Their data also indicated that sperm chromatin damage was not age- or histotype-dependent, but was more marked in advanced stages of TC and was also influenced by the type and intensity of treatment.

**Table 2:** Post-treatment sperm DNA quality in testicular cancer patients subdivided by method used.

**References**	**N. TC pts**	**Follow-up**	**Treatment**	**DNA integrity**
**SCSA**				
Stahl et al. (114)	74	0, 6, 12, 24, 36, and 60 months	Chemo- and Radiotherapy	RT: increased DFI up to 2 years post-treatment; normalization after 3–5 years CH: reduced DFI up to 5 years post-treatment
Stahl et al. (115)	96	0, 6, 12, 24, 36, and 60 months	Chemo- and Radiotherapy	RT: increased DFI up to 2 years post-treatment; normalization after 3–5 years CH: reduced DFI up to 5 years post-treatment
Stahl et al. (116)	58	Mean 3 years	Chemo- and Radiotherapy	No differences in DFI pre- and post- treatment—DNA not affected by treatment
Smit et al. (118)	52	Range 0.5 to 3.3 years	Chemo- and Radiotherapy	RT: increased DFI against CH (mean 1.1 years)
O'Flaherty et al. (119)	16	0, 6, 12, 18, and 24 months	Chemotherapy	CH: increase in SD DFI and HDS inTC patients up to 24 months post-therapy.
Bujan et al. (121)	53	3, 6, 12, and 24 months	Chemo- and Radiotherapy	RT: reduced chromatin compaction to T6 post RT CH: no DFI variation pre- and post-CH
Paoli et al. (43)	254	3, 6, 9, 12, and 24 months	Chemo- and Radiotherapy	RT: increased DFI at 3 and 6 months, less marked reduction at 12 and 24 against CH CH: increased DFI at 3 and 6 months and reduction at 12–24 months
**TUNEL**				
Stahl et al. (115)	96	0, 6, 12, 24, 36, and 60 months	Chemo- and Radiotherapy	RT: increased DFI up to 2 years post- treatment; normalization after 3–5 years CH: reduced DFI up to 5 years post-treatment
Spermon et al. (123)	22	Range 18.4–84.8 months	Chemotherapy	Chromatin condensation improved after treatment DNA fragmentation not reduced after CH
Bujan et al. (121)	53	3, 6, 12, and 24 months	Chemo- and Radiotherapy	No change in sperm DNA fragmentation pre- and post-treatment
Ghezzi et al. (138)	212	0, 12, 24 months	100 BEP 54 CARB 58 surveillance	BEP: at 12 and 24 months increased post therapy DNA damage both vs. baseline and vs. CARB.
**COMET**				
O'Donovan (122)	13	0, 3, 6, 12 months	Various antineoplastic agents	Reduced percentage of intact sperm DNA and chromatin condensation
O'Flaherty et al. (126)	16	0, 6, 12, 18, and 24 months	Chemotherapy	Increased sperm DNA fragmentation 6 months post-treatment against T0, remaining elevated up to 18–24 months

*RT, radiotherapy; CH, chemotherapy; BEP, Bleomycin, etoposide, cisplatin; CARB, carboplatin*.

#### TUNEL

Several studies used TUNEL to investigate the impact of chemotherapy and radiotherapy on TC patients (Table 2). Stahl et al. confirmed the results they had seen with SCSA, finding increased sperm DNA damage for 2 years post-RT with both methods, thus indicating a correlation between the two (115). Bujan et al. did not find any increased fragmentation after CT or RT (121). Spermon et al. who investigated the effects of 4 cycles of BEP in a small caseload of TC patients against normozoospermic subjects after a mean of 48.2 months, achieved similar results (123). These authors did not find any difference between sperm DNA fragmentation pre- and post-chemotherapy, but only against controls. Although sperm nuclear quality did not reach normal levels, semen samples did show improved chromatin condensation. In contrast, Ghezzi et al. compared patients treated with BEP, carboplatin or under surveillance alone, finding that BEP caused significantly more DNA damage than one cycle carboplatin. This damage was still detectable in BEP patients after 24 months of follow up when compared to baseline values. This seems to reflect the fact that more intensive chemotherapies might have higher influence on DNA integrity and for longer time (138).

#### Comet assay

In 2005, O'Donovan evaluated chromatin integrity pre- and post-antineoplastic therapy in various neoplastic diseases including testicular cancer (122). This was a pilot study on a small caseload, of whom just 13 had TC. There was a lower percentage of intact sperm DNA (percentage head DNA intact) and chromatin condensation after treatment in patients than in the controls. O'Flaherty et al. obtained different results in a study of 16 post-orchiectomy TC patients who underwent BEP compared to healthy male volunteers (126). This longitudinal study found that chemotherapy has a negative impact in testicular cancer patients, with increased sperm DNA fragmentation 6 months after the end of treatment in comparison with the baseline; this value was still raised at 18–24 months. This study thus demonstrated that chemotherapy can induce long-lasting DNA damage.

In summary, a total of 11 papers used various methods to evaluate the impact of chemo- and radiotherapy on sperm DNA integrity in testicular cancer patients. Some of these used more than one method (Table 2), with 5 using SCSA, 2 TUNEL, 2 Comet and 2 studies used both SCSA and TUNEL to investigate the same caseload. We identified just one paper which did not find any sperm DNA damage in TC patients after chemo- or radiotherapy, but the author himself suggested that the high inter-subject variation in the impact of the antineoplastic treatment on chromatin integrity could affect the results. Furthermore, the median post-therapy observation time was about 3 years in this study (range 1–20 years) and the lack of information on DNA integrity shortly after treatment could cause the real impact of the therapies to be underestimated (116). In contrast, most studies found sperm DNA to be more sensitive to radiotherapy, which induces transient damage from 6 months after the end of the treatment but normalizes within 2–5 years. In fact, radiation induces material ionization both directly, through excitation of the atoms making up the DNA molecule, and indirectly, through its interaction with non-DNA molecules, which induce the ionization of the genetic material by emitting secondary electrons (95, 139).

Evidence for the genotoxic effect of chemotherapy on sperm is less clear-cut than that for radiotherapy. Many chemotherapeutic drugs penetrate the Sertoli cell barrier and damage germ cells. Type B spermatogonia, which proliferate actively, are extremely susceptible to cytotoxic agents; chemically stable DNA adducts produced in the testicles can persist, inducing DNA strand breakage during the spermatogenic process. However, type A spermatogonia, which have little mitotic activity, are less affected and could survive polychemotherapy if threshold cumulative cytostatic doses are not surpassed (140).

Two studies found reduced damage after chemotherapy (114, 115), suggesting that spermatogonia with abnormal chromatin arising from defective DNA repair mechanisms might be more sensitive to chemotherapy (115). However, it should be stressed that these studies involved relatively small caseloads, especially after stratification by time since end of treatment and by type of antineoplastic treatment. Other studies found that chemotherapy had a negative impact on the sperm chromatin profile lasting up to 2 years after the end of the therapy. Various components of the chromatin structure may be modified by different chemotherapeutic agents, thus affecting not only fragmentation but also chromatin compaction (119). The different components of the chromatin may thus take different times to be repaired after chemotherapy and the extent of sperm DNA damage may also differ. It should also be noted that studies evaluating pre- and post-therapy chromatin integrity are highly heterogeneous and the results could be affected not only by the small caseloads but also by the different methods used. TUNEL and alkaline Comet measure the number of double or single DNA strand breaks, while SCSA provides an indirect measure of DNA damage. The different recovery times could also depend on the different follow-up times used in the various studies, given that they did not all analyse samples at the same times. Furthermore, the literature analyzed to date reveals that sperm chromatin damage is not age- or histotype-dependent, but appears more marked in advanced stages of TC as well as being influenced by the type and intensity of treatment. This could explain the divergent results on the impact of chemotherapy on sperm DNA integrity obtained by the different authors discussed above, and more studies are needed to provide conclusive evidence.

## Paternity and testicular cancer

The paternity of TC survivors is a particularly interesting topic. These patients often ask about their chance of fatherhood, the teratogenicity of their treatment or the risk of TC in their offspring. Around 40% of patients pre-diagnosis and 50% post-diagnosis want children (141). For this reason, the cryopreservation of semen and/or testicular tissue is of fundamental importance in the clinical management of this disease (142, 143). Various antineoplastic treatments can induce mutagenic effects in both somatic cells and male germ cells at various stages of maturation. However, we are as yet unable to predict the extent of any genomic damage, the potential teratogenic effect and the long term effects on fertility and offspring outcome caused by these treatments. Studies in mouse models have shown that cisplatin induces chromatid breaks and fragments in spermatocytes and spermatogonia immediately after treatment, whereas conflicting results have been reported in relation to diploidy and disomy in treated patients (135, 144–146). The injurious effect of chemotherapy on offspring may be related to abnormal sperm chromatin structure. Proteome studies (147) found up-regulation of histones and a significant decrease in protamine in the mouse sperm head, while other studies found increased methylation in drug-treated animals (148, 149).

Although sperm chromatin damage has been observed to a greater or lesser extent in TC patients, it does not seem to be invariably correlated with a diagnosis of infertility in the survivors of this disease. Sperm DNA mutations seem to be associated with a lower fertility than found in the healthy population and an increased risk of early abortion. However, there is a lack of prospective studies in the literature investigating the correlation between TC-induced sperm DNA damage and reproductive outcome. This would be extremely useful information, especially as the ever-increasing use of ART could in theory give rise to a greater risk of selecting sperm with damaged DNA. The available data on the *in vivo* and *in vitro* reproductive capacity of TC survivors and the known effects on offspring are discussed below.

### Data from national registers

Population studies, mainly originating from north European national registers, suggest that the fertility of cancer survivors is lower than that of the general population. Fifty percent of post-cancer patients presented primary infertility (150). Fossa et al. reported that on average, cancer patients had conceived at least one child 3–4.5 years after the end of the treatment (151). In comparison with the general population, testicular cancer survivors had a 33% lower probability of having a child within 5 years after diagnosis and 20% lower after more than 5 years (152); these data were confirmed by later studies (150, 153–155).

The most recent observation comes from a study by Gunnes et al. who found in data from Norwegian registers a reduced probability of fatherhood (HR 0.77) and greater use of ART in TC survivors than in the control population (155). It should be stressed that these population studies have limitations. Studies based on caseloads from national registers often lack details of the type of treatment, the physical condition of individual patients, the physical condition of neonates after the first day of life and information on the female partner such as maternal age, parity, smoking, and behavior during pregnancy and, above all, many of these studies do not specify if pregnancy occurred naturally or following ART. On the whole, they demonstrate that male cancer survivors, including TC survivors, do have a chance of achieving fatherhood through natural conception or assisted reproduction despite the damage induced by the antineoplastic treatment, even if this chance is lower than in the general population.

### Effects on offspring

The incidence of any effects in the offspring of fathers treated with antineoplastic therapies was not always investigated by the various studies and the evidence in the literature is highly contradictory. Unlike in children born to mothers with a previous diagnosis of cancer, some Danish multicentre and register studies did not find any increased risk of congenital or genetic abnormalities in the children of male cancer survivors treated with chemotherapy or radiotherapy (151, 152, 156–158) and did not find any increased risk of perinatal death, low birth weight or preterm birth (155). In contrast, Magelssen et al. found an increased incidence of congenital abnormalities in firstborn infants fathered after the cancer diagnosis, regardless of whether or not ART was used (153). The abnormalities reported in this study were observed after various types of treatment and up to 15–20 years after diagnosis, even if they were not correlated with any specific antineoplastic treatment. Data from Danish and Swedish registers also found that children with a paternal history of cancer had a significantly increased risk of any congenital abnormality (RR = 1.12, CI 95% 1.02–1.24; *p* = 0.018), and especially major congenital abnormalities (RR = 1.17, CI 95% 1.05–1.31; *p* = 0.004), regardless of how they were conceived (natural, ART, cryopreserved, or fresh semen), than the children of healthy controls (159). This risk was higher among children born within 2 years of their father's cancer diagnosis, suggesting that the cause is the transient effect of treatment on sperm DNA quality. In this case too, interpretation of the data is limited by the nature of the registers; although they report events in very large caseloads, they cannot unequivocally trace such events back to the impact of previous antineoplastic chemo- or radiotherapy.

Given that measurable sperm nuclear damage has been demonstrated for both RT and CT, especially in the first 12–24 months after the end of treatment, it is reasonable to suppose that malformations and early abortions occurring in this time period can probably be attributed to the effects of the treatment on sperm DNA. This aspect should thus be discussed openly with the patient, to enable the protection of the patient's future fertility through cryopreservation of semen or testicular tissue before beginning any treatment (Table 3A).

**Table 3A:** Summary of available data from national registers/population studies on fertility in testicular cancer survivors.

**Reference**	**Total male cancer patients**	**Fatherhood after diagnosis**	**Total use of ART**	**Testicular cancer patients (%)**	**Children after TC diagnosis (%)**	**Probability of fatherhood**	**Risk of major congenital malformations in offspring**
Fossa et al. (151)	5,173	972	N/A	1854 (35.8%)	429 (23.1%)	8 and 14% after 5 and 10 years	No increased risk
Syse et al. (152)	7,127	1,731	N/A	567 (7.9%)	N/A	OR 0.8 after 5 years^b^	N/A
Madanat et al. (150)	11,985	1,834	N/A	1,273 (10.6%)	366 (28.7%)	RR 0.57 for first child^c^	N/A
Magelssen et al. (153)	463	142	8.4%	211 (45.6%)	72 (34.1%)	42% 10 years post-diagnosis^d^	27 cases reported^a^ OR = 1.8^b^
Stahl et al. (159)	N/A	8,670	5.9%	N/A	N/A	N/A	Increased risk RR = 1.17^a^
Stensheim et al. (154)	11,451	2,618	2.3%	3,511 (30.7%)	1,081 (30.8%)	HR 0.74^a^	N/A
Signorello et al. (156)	1,128^e^	1,128^e^	N/A	None	N/A	N/A	36 cases reported. no increased risk^a^
Winther et al. (157)	722	722^e^	N/A	N/A	N/A	N/A	No increased risk^a^
Stensheim et al. (158)	2,087	2,087^e^	2.6%	805 (38.6%)	N/A	N/A	No increased risk^a^
Gunnes et al. (155)	2,687	1,087	3.0%	734 (27.3%)	349 (47.5%)	HR 0.77^a^	No increased risk^a^

### Data on natural fertility

Various studies have investigated natural fertility pre- and post-treatment in TC survivors, with a broad range of results. Some studies reported that 39 and 40% of TC patients achieved paternity through natural means before starting any treatment (160, 161) while others found a much higher pre-therapy fertility rate of between 77.5 and 91% (141, 162, 163). This suggests that, at least in a subgroup of patients, fertility may already be compromised at the baseline.

After treatment, 52% of TC patients fathered a child, most within 1 year (161). A natural pregnancy was achieved by 64% of seminoma patients treated with radiotherapy with testicular shielding (164), although other authors (163) found that radiotherapy was more harmful than chemotherapy, and cumulative conception rates for patients treated with radiotherapy were significantly lower than the rates for patients treated with chemotherapy. Brydoy et al. found that after 2–4 cisplatin-based chemotherapy cycles around 80% (85/106) of TC patients fathered a child by natural conception and the probability of fatherhood was inversely correlated with the number of cycles (165). However, Matos et al. reported lower natural post-therapy fertility rates, at 50% (74/150) (141). In Ping et al.'s study, 21.9% (16/73) had conceived naturally and 26.0% (19/73) by ART (166). The literature thus contains a wide range of data, probably due to differences in irradiation procedures and chemotherapy regimens; for this reason, as well as differences in the patient cohorts and the aims of each study, the comparison of different studies is difficult.

### Data on ART

TC survivors seem to resort to ART more frequently than the general population, with around three times the number of pregnancies resulting from ART (154, 155). Lass et al. hypothesized that the improvement in treatments and in the life expectancy of cancer patients would lead to a greater number of patients being offered the chance of semen cryopreservation for use in ART (167). Kelleher et al. studied 833 patients with different types of cancer (37% with TC) who had cryopreserved their semen, finding that 7.7% (64 patients) had used it for various assisted reproduction techniques, achieving a total of 29 pregnancies (168). In contrast, Chung et al., who also studied patients with different types of cancer (including 42 with TC), reported that just 4.7% (6/127 patients) had used cryopreserved semen, resulting in two pregnancies, one by IVF and one using ICSI (169). Agarwal et al. (170) and Schmidt et al. (171) found higher success rates with ART, with respectively 4/9 TC patients and 34/67 cancer patients (predominantly TC and Hodgkin's disease) achieving at least one child born following ART with fresh or frozen semen. Magelssen et al. reported that 14/29 TC patients had conceived at least one healthy child, while two pregnancies had ended in spontaneous abortion (172). Later studies found excellent pregnancy rates (above 50%) in the partners of patients who had undergone semen cryopreservation for various cancers, especially TC (173, 174). In contrast, Crha et al. reported a somewhat lower success rate, at 29.5% (175).

Ping et al. (176), Freour et al. (177), and Bizet et al. (178) all studied large caseloads of post-treatment cancer patients, but these included few TC patients. These authors reported a lower pregnancy rate following ART. Botchan et al. reported the use of ART in 70/682 patients who had cryobanked semen prior to cancer treatment, achieving 36 to-term pregnancies (179). The previously cited study by Ping et al. (166) analyzed the reproductive outcome of 117 TC patients post-treatment, finding that 19 (26%) of the 73 patients attempting fatherhood had been successful through ART with fresh semen (11 patients) or cryobanked semen (8 patients), similar to the results of a later study by Gil et al. (180). Zakova et al. (181) found a higher pregnancy rate, at 47% (16/34 patients), and better results were also reported by Molnar et al. (182) (57%; 4/7 patients). Sonnenburg et al. (183) reported live births with ART in 82% (9/11) of the couples that used banked semen, while Garcia et al. (184) found live birth rates in cancer patients and cancer-free infertile patients similar to the previous studies (Table 3B). Finally, in a recent systematic review, Ferrari et al. (185) reported that, with a relatively low use (8%) of cryobanked semen samples, the cumulative percentage of couples achieving pregnancy by ART was around 49%.

**Table 3B:** Summary of available data from cohort studies on natural and ART fertility in testicular cancer survivors.

**Reference**	**Total male cancer patients**	**Total use of ART**	**Testicular cancer patients**	**Children before TC diagnosis (%)**	**Children after TC diagnosis (%)**	**Children after ART in TC patients**	**Time to first child after TC diagnosis**	**Adverse events in offspring**
Hartmann et al. (160)	98	N/A	98	39 (39.8%)	21 (21.4%)	N/A	Median 54 months after treatment	None reported, 3 miscarriages
Kelleher et al. (168)	901	64 (7.7%)	348	N/A	N/A	N/A	Median 36 months	3 cases reported^a^
Spermon et al. (162)	226		226	93/120 (77.5%)^f^	54/88 (61.4%)^f^	8/88 (14.8%)	Median 46 months	None reported, 7 miscarriages
Agarwal et al. (170)	31^g^	12 (38.7%)	11	N/A	8 (72.7%)	6 (54.5%)	N/A	None reported, 4 miscarriages^a^
Chung et al. (169)	164^g^	6/127 (4.7%)^f^	42	N/A	N/A	1/3 (33.3%)^f^	N/A	N/A
Huyghe et al. (163)	446		446	208/228 (91.2%)^f^	110/164 (67.1%)^f^	7/164 (6.4%)	Mean 4.8 ± 3.0 years	N/A
Nalesnik et al. (164)	73		73	N/A	6/11 (54%)^f^	N/A	N/A	None reported, 1 miscarriage
Schmidt et al. (171)	67	34 (50.7%)	34	N/A	N/A	N/A	N/A	7 miscarriages, 2 ectopic^a^
Magelssen et al. (172)	1,388		1,388	693 (49.9%)	513 (39.0%)^h^	29 (2.1%)	N/A	2 spontaneous abortions after ART
Girasole et al. (161)	129		129	65 (50.4%)	28 (21.7%)	2 (7.1%)	N/A	N/A
Hourvitz et al. (174)	118	118 (100%)	47	N/A	N/A	N/A	N/A	11 spontaneous abortions
van Casteren et al. (173)	629	18/37 (48.7%)^f^	236	N/A	N/A	N/A	N/A	2 early abortions after ART^a^
Chra et al. (175)	619	28 (4.5%)	270	N/A	N/A	N/A	Median 18 months	N/A
Ping et al. (176)	1548^i^	2/30 (6.7%)	21	N/A	1/30 (3.3%)	1/30 (3.3%)	N/A	N/A
Matos et al. (141)	297		297	98/119 (82.4%)^f^	74/150 (49.3%)^f^	N/A	Median 12 years from diagnosis	N/A
Freour et al. (177)	1042	82 (7.9%)	438	N/A	2/27 (7.4%)^f^	2/27 (7.4%)^f^	N/A	None reported
Bizet et al. (178)								
Botchan et al. (179)	682	27/70 (38.6%)^f^	216	N/A	N/A	13/18 (72.2%)^f^	63% tried ART within 3 years	None reported, 4 abortions, 3 extra-uterine
Molnar et al. (182)	52		52	N/A	16 (30.8%)	4/7 (57%)^f^	N/A	6 miscarriages reported
Ping et al. (166)	117		117	21/69 (30.4%)^f^	35/73 (47.9%)^f^	19/73 (26.0%)^f^	N/A	None reported, 4 miscarriages
Zakova et al. (181)	523	16/34 (47%)^f^	523	N/A	N/A	16/34 (47%)^f^	ART about 18 months after semen cryopreservation	N/A
Garcia et al. (184)	272	14/29 (48.2%)^f^	52	N/A	N/A	9 (17.3%)^f^	N/A	N/A

a *All types of cancer*.

*^b^Compared to control population*.

*^c^Compared to siblings without cancer*.

*^d^Testicular cancers*.

e *Only patients with children were selected*.

f *Fatherhood achieved/patients who tried to conceive*.

g *Only patients who cryopreserved their semen*.

h *Patients with children 20 years after diagnosis*.

i *Only 30 cancer patients*.

All these studies show that ART is a valid option for patients who are unable to achieve a natural pregnancy. Although some authors have hypothesized an additional risk of adverse effects in the offspring after ART in these patients, this suspicion must be confirmed by further study (186). The limitations of these studies include the diversity of their caseloads and the different ART methods used, which could influence the published results. In fact, although some caseloads were relatively large, they generally included survivors of different types of cancer in different organs, which could affect the reproductive axis (and hence fertility) in different ways. Furthermore, it is not always possible to extrapolate the data for TC patients alone, who in some cases amount to just a few individuals. Finally, since the oocyte has an essential role in maintaining genome integrity and in reducing the transmission of new mutations and chromosomal structural aberrations to the offspring (187–189), another confounding factor is that the clinical data of the female partners, which could influence the outcome of ART, is often unreported.

## Conclusions

### Pre-therapy sperm DNA damage

Most papers found a high level of sperm DNA damage in TC patients in comparison with healthy, fertile controls but no significant difference between TC patients and infertile patients. However, it should be stressed that these studies have various limitations which affect the comparison of the cases and controls and make it difficult to interpret the results.

### Post-therapy sperm DNA damage

Some literature reports of treatment-induced sperm DNA changes indicated increased chromatin damage for up to 2 years after the end of the treatment. Such damage is more marked in advanced stages, suggesting that sperm from patients with more invasive TC is more vulnerable to antineoplastic treatments.

Post-therapy DNA damage is also influenced by the treatment type and dose. The spermatogenic line seems to be more sensitive to radiotherapy than to chemotherapy. However, the various studies do identify negative effects of both TC and its treatments on the DNA structure which could affect the reproductive capacity of TC patients.

All these aspects should be discussed with the patient during counseling before beginning any potentially genotoxic treatment, to enable the protection of the patient's future fertility, if desired, through cryopreservation of undamaged semen or testicular tissue.

In any case, additional studies with a greater statistical power are needed to confirm the effects and persistence of sperm DNA damage. Specifically, further characterization of the type of damage seems necessary, in order to establish more precisely its correlation with reproductive outcomes.

### Fatherhood

Population studies reveal that north European cancer survivors have an overall reproductive capacity about 24–30% lower than that of the general population, regardless of the tumor type. Some studies found that TC survivors had a 33% lower probability of having a child within 5 years after diagnosis and 20% lower after more than 5 years. The main limitation of these studies is that they investigate cancer registers. Although these registers enable large caseloads to be studied, they include all types of cancer, and it is not always possible to extrapolate data for TC survivors alone: data pertaining to the fertility of these subjects vary considerably according to the tumor site. Furthermore, they do not include important information which might influence both patient and couple fertility, such as type of treatment, physical condition of individual patients and of the neonates, and information on the female partner such as maternal age, parity, smoking, and behavior during pregnancy.

Studies of cohorts of TC survivors report conflicting evidence in relation to natural paternity, with rates ranging from 20 to 80%. Moreover, although they demonstrate that paternity for TC patients is possible, these studies are difficult to compare due to their inclusion of different treatment types, age of onset, and above all sample size. TC survivors seem to resort to ART more frequently than the general population, with around three times the number of pregnancies resulting from ART. In fact, ART has increased the chance of fatherhood for these patients, including through the use of cryobanked sperm. The studies included in this review demonstrate a success rate of around 50%, especially with more advanced techniques (ICSI); again, however, their analysis is limited by their different treatment types and the inconsistent presence of data on the female partner.

### Congenital anomalies

There are contradictory reports of the incidence of any effects in the offspring of fathers treated with antineoplastic therapies. Most authors did not find any increased risk of congenital or genetic abnormalities, perinatal death, low birth weight or preterm birth in the children of male cancer survivors treated with chemotherapy, or radiotherapy. However, others reported an increased risk of congenital abnormalities, at their peak in children born within 2 years of their father's cancer diagnosis. This suggests that they are caused by the effect of the treatment on sperm DNA quality, as highlighted by sperm chromatin studies (Table 4).

**Table 5:** Summary of evidence and future suggestions.

Sperm DNA damage	Higher pre-treatment sperm DNA damage in TC patients in comparison with healthy, fertile controls but without significant differences between TC and infertile patients. Sperm DNA damage detected for up to 2 years after the end of the treatment. Such damage is more marked in advanced stages and is also influenced by the treatment type and dose.
Fatherhood chance	*Population studies* report a reduced (about 25% lower) reproductive capacity in cancer survivors than in the general population, regardless of the tumor type. *Studies of cohorts* of TC survivors report a wide range of natural paternity rates (20–80%). However, due to heterogeneity, these data are difficult to evaluate. TC survivors resort to ART more frequently than the general population. ART, including the use of cryobanked sperm, has a success rate of around 50%, especially with more advanced techniques (ICSI).
Congenital anomalies	Most authors did not find any increased risk of major anomalies (congenital or genetic abnormalities, perinatal death, low birth weight, preterm birth) in the children of male cancer survivors treated with chemotherapy or radiotherapy. A few authors found an increased risk of congenital abnormalities, at their peak in children born within 2 years of their father's cancer diagnosis. Several authors reported miscarriages, but did not compare rates against cancer-free patients.
Future suggestions	Negative effects on sperm DNA structure which may affect the reproductive capacity of TC patients make adequate counseling essential before beginning any potentially genotoxic treatment. Clinicians should also discuss post-treatment fertility as well as sperm cryopreservation strategies and the possible future use of ART. More high quality studies with adequate follow ups are needed to confirm previous observations of sperm DNA damage. Further studies investigating the extent of this damage might identify the time needed for the germ cell line to repair nuclear damage and thus produce gametes with a reduced risk for the offspring.

In conclusion, oncofertility is a highly interesting field, especially in TC survivors, as the marked improvement in prognosis in recent decades has caused attention to shift to quality of life and reproductive health. Sperm chromatin damage associated with genome instability is the most important reproductive side effect related to the malignancy or its treatment. Studies investigating the extent of this damage could have a considerable translational importance in the management of cancer patients, as they could identify the time needed for the germ cell line to repair nuclear damage and thus produce gametes with a reduced risk for the offspring. However, given the ambiguity of the results of the studies reported in the literature to date, further studies with a greater statistical power are needed.

## Author contributions

DP and FP conceived the work, identified the articles, and wrote the paper. FL and AL revised the paper critically and gave final approval. All authors read and approved the final manuscript.

## Funding

This work was supported by a grant from the Italian Ministry of Education and Research (MIUR-PRIN 2015- 2015XSNA83-002) and the University of Rome La Sapienza Faculty of Medicine.

### Conflict of interest statement

The authors declare that the research was conducted in the absence of any commercial or financial relationships that could be construed as a potential conflict of interest. The reviewer PC and handling Editor declared their shared affiliation.
